# DNA Builds and Strengthens the Extracellular Matrix in *Myxococcus xanthus* Biofilms by Interacting with Exopolysaccharides

**DOI:** 10.1371/journal.pone.0051905

**Published:** 2012-12-26

**Authors:** Wei Hu, Lina Li, Shivani Sharma, Jing Wang, Ian McHardy, Renate Lux, Zhe Yang, Xuesong He, James K. Gimzewski, Yuezhong Li, Wenyuan Shi

**Affiliations:** 1 State Key Laboratory of Microbial Technology, School of Life Science, Shandong University, Jinan, Shandong, China; 2 School of Dentistry and Molecular Biology Institute, UCLA, Los Angeles, California, United States of America; 3 Department of Chemistry and Biochemistry and California NanoSystems Institute (CNSI), UCLA, Los Angeles, California, United States of America; 4 International Center for Materials Nanoarchitectonics Satellite (MANA), National Institute for Materials Science (NIMS), Tsukuba, Japan; The University of Hong Kong, Hong Kong

## Abstract

One intriguing discovery in modern microbiology is the extensive presence of extracellular DNA (eDNA) within biofilms of various bacterial species. Although several biological functions have been suggested for eDNA, including involvement in biofilm formation, the detailed mechanism of eDNA integration into biofilm architecture is still poorly understood. In the biofilms formed by *Myxococcus xanthus,* a Gram-negative soil bacterium with complex morphogenesis and social behaviors, DNA was found within both extracted and native extracellular matrices (ECM). Further examination revealed that these eDNA molecules formed well organized structures that were similar in appearance to the organization of exopolysaccharides (EPS) in ECM. Biochemical and image analyses confirmed that eDNA bound to and colocalized with EPS within the ECM of starvation biofilms and fruiting bodies. In addition, ECM containing eDNA exhibited greater physical strength and biological stress resistance compared to DNase I treated ECM. Taken together, these findings demonstrate that DNA interacts with EPS and strengthens biofilm structures in *M. xanthus*.

## Introduction

Natural biofilms, a preferred lifestyle of many bacteria, consist of highly structured polymer-encased communities [1]. They differ profoundly from their planktonic counterparts in physiological responses and developmental dynamics [2]. They also enable bacteria to grow on surfaces in a self-produced matrix, and provide a mechanically stable and protective environment [3]. Among the most intricate biofilms is the one formed by *Myxococcus xanthus* [4], [5], a motile Gram-negative soil bacterium that employs gliding motility in coordinated efforts to accomplish the various tasks of its complex life cycle [6]. Under starvation conditions, *M. xanthus* cells initiate and undergo a developmental process culminating in the formation of a multicellular fruiting body filled with myxospores [7]. By controlling the concentration of Ca^2+^ in submerged cultures, highly-organized developmental biofilms (fruiting bodies) and simple non-developmental starvation biofilms of *M. xanthus* can both be cultivated [8]. Based on this ability to control the type of formed biofilm, *M. xanthus* might serve as a versatile model organism in biofilm research.

Since bacterial biofilms play important roles in the pathogenesis of many chronic infections [9], it is essential to understand in detail the process of biofilm formation and the roles, interactions and composition of the various matrix components within the polymeric biofilm architecture. The extracellular matrix is defined as a mixture of exopolysaccharides (EPS), proteins, nucleic acids and other components released from cells that act as a physical substratum to maintain biofilm structure and functions [10], [11]. In *M. xanthus* biofilms, the ECM (also referred to as slime layer or fibrils [12]) is distributed over the entire cellular surface [13] and connect neighboring cells to each other as well as to the surface [14]. A biochemical analysis of purified ECM identified a carbohydrate EPS matrix with associated proteins, and subsequent compositional analysis established the presence of five different monosaccharides in the EPS [15]. In addition to EPS, protein and LPS, DNA molecules may also be partially sequestered in the intercellular spaces and within the slime layer (ECM) [16]. However, DNA presence and involvement in the formation of biofilms of *M. xanthus* remains unclear.

Extracellular DNA (eDNA) has been found in biofilms of a number of bacterial species, and is considered a functional ECM component. eDNA has been reported as: a source of genetic material for horizontal gene transfer [17], a source of nutrients for live cells [18], [19], a buffer that can recruit/titrate ions or antibiotics [1], [20], a factor to inhibit the attachment and promote the dispersal of a specific cell type in a biofilm [21], and a contributor to biofilm formation [1], [22], [23], [24], [25], [26]. However, the biological relevance, the mechanism by which eDNA is integrated into biofilms, and how it interacts with other macromolecules in the matrix, have not been well established. In this study, we propose that the eDNA molecules participate in establishing the architecture of *M. xanthus* biofilms by interacting directly with EPS molecules. These interactions contribute to the construction of an integrated ECM with enhanced physical strength and resistance to biological stresses.

## Materials and Methods

### Bacterial Strains, Media and Growth Conditions

*M. xanthus* wild-type strain DK1622 [27] cells were grown in CYE medium [28] at 32°C on a rotary shaker at 300 rpm. To cultivate biofilms under starvation conditions, exponentially growing cells were harvested, vortexed in the presence of 3 mm glass beads to disrupt cell clumps, and washed 3 times with MOPS buffer (10 mM MOPS, 8 mM MgSO_4_, pH 7.6). The cells were then resuspended in MOPS buffer to 5×10^8^ cells/ml and incubated to form non-developmental starvation biofilms in sealed containers at 32°C in the dark. After 24 hr, a thin layer of typical biofilm structure formed on the bottom of 8-well chambers (Lab-Tek II Chamber Slide System, Nalge Nunc, USA) that had been modified [29] by replacing the bottom slides with cover slips for observation with a confocal laser scanning microscope (CLSM). For submerged fruiting body culture conditions, cells were prepared as described above, diluted to 2.5×10^7^ cells/ml in fresh CYE medium and incubated at 32°C for 24 hr. CYE medium was then gently and completely removed by aspiration and then replenished with the same volume of MMC buffer (10 mM MOPS, 4 mM MgS0_4_, 2 mM CaCl_2_, pH 7.6) to induce starvation conditions [8]. The cells were then incubated in sealed containers at 32°C in the dark. The initial fruiting body structures were formed at 24 hr and more mature fruiting bodies could be observed in the 48 hr aggregates. Samples were kept in a humidity chamber for extended incubation periods to prevent desiccation.

### Isolation of Chromosomal DNA, EPS and Cell-free Extracellular Materials of *M. xanthus*

Chromosomal DNA of DK1622 strains was prepared as described previously [30], and the concentration was determined with a Quant-iT DNA assay kit (Invitrogen, USA) using a fluorometer (VersaFluor, BioRad, USA) with 500 nm excitation and 520 nm emission filters.

Protein-free EPS was isolated from DK1622 as previously described [31], [32], with the following modifications to generate nucleic acid-free EPS: Before pronase (Sigma, USA) treatment, 200 µg/ml of DNase I and RNase (Worthington, USA) were added to the EPS sample (5 mg/ml) at 37°C and incubated for 24 hr. The Sevag assay [33] was employed to remove the remaining proteins from the EPS sample. Samples were then lyophilized, a portion of the lyophilized EPS sample was resuspended in deionized water and analyzed for purity. The DNA content in the isolated EPS was determined with a Quant-iT high sensitivity DNA assay kit (Invitrogen), the protein content was determined with a Pierce BCA protein assay kit (Thermo Scientific, USA) and the carbohydrate content was determined by the anthrone assay [34].

**Figure 1:**
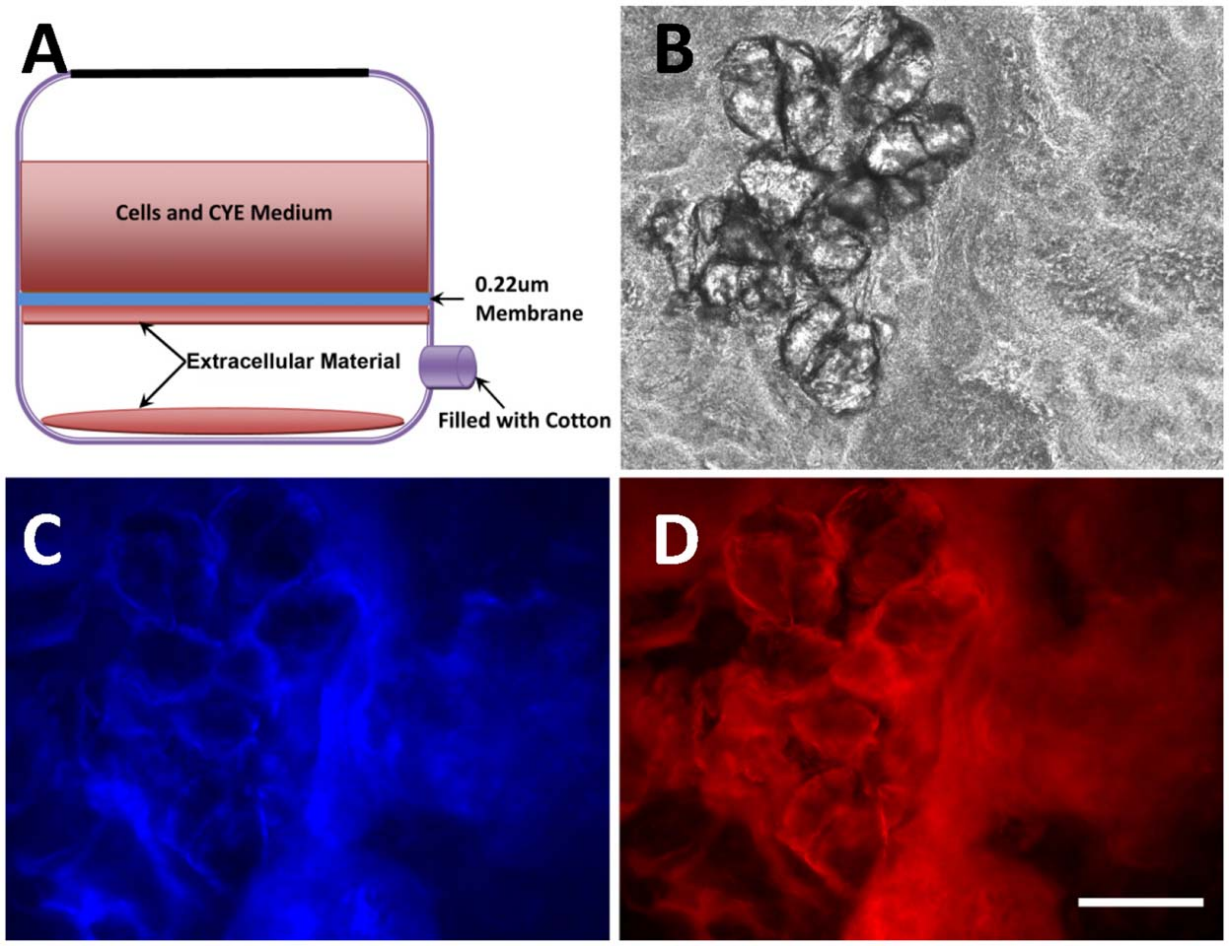
Isolated cell-free extracellular materials from DK10547 by a membrane-chamber system contained both EPS and eDNA. Schematic diagram of the 150 ml membrane-chamber system used to isolate cell-free extracellular materials (panel A). Through an up-right fluorescence microscope (panel B, normal phase image), EPS and eDNA were detected in the isolated extracellular materials by calcoflour white staining (panel C) and PI staining (panel D), respectively. The bar represents 100 µm.

A membrane-chamber system (Fig. 1A, based on the 150 ml Stericup system, Millipore, USA) was developed to isolate cell-free extracellular materials from *M. xanthus* strain DK10547, a *gfp*-expressing derivative of strain DK1622 [35]. 5×10^9^ cells were inoculated in 100 ml fresh CYE liquid medium in the upper chamber of a filter, containing a 0.22 µm porous membrane, and incubated at 32°C for 96 hr. During the incubation, fresh CYE liquid medium was replenished in the upper chamber to maintain the broth volume at 100 ml. The contents of the bottom-chamber were subjected to 17,000×*g* centrifugation for 60 min at 4°C to collect the cell-free extracellular materials which was mainly composed of ECM.

### Sample Preparation and Confocal Laser Scanning Microscopy

Confocal laser scanning microscopy (CLSM) was employed to analyze *M. xanthus* starvation biofilm and fruiting body structures with various dye combinations [29] using a PASCAL 5 confocal laser scanning microscope (Zeiss, Germany). The scanning module of the system was mounted on an inverted microscope (Axiovert 200 M) and samples were viewed through a 40× oil-immersion objective (Plan-Neofluar/NA 1.3) or a 63× oil-immersion objective (Plan-Apochromat/NA1.4). Excitation at 488 nm with an argon laser in combination with a 505–530 nm band-pass emission filter was used for GFP and SYTO 9 fluorescence imaging. SYTOX orange signals were visualized using 543 nm excitation with a helium–neon laser and a 560–615 nm band-pass emission filter. Excitation at 543 nm with a helium–neon laser and a 650 nm long-pass emission filter was used to reveal FM 4-64 signals. Excitation at 633 nm with a helium–neon laser and a 650 nm long-pass emission filter was used to observe Alexa 633 signals.

### Staining of Nucleic Acid, EPS and Cell Membranes

Propidium iodide (PI), a specific DNA binding dye (Molecular Probes, USA), was used to stain the DNA in isolated cell-free extracellular materials at 5 µM. Samples were then subjected to fluorescent microscopy (Nikon E-400, Japan) with 543 nm excitation. A combination of 5 µM SYTO 9 (Invitrogen), a green fluorescent cell membrane permeable nucleic acid binding dye, and 10 µM SYTOX orange (Invitrogen), a red fluorescent cell membrane impermeable nucleic acid binding dye was used in the CLSM experiments. The presence of SYTOX orange causes a reduction in the SYTO 9 stain fluorescence, which allows *M. xanthus* with intact cell membranes to be stained with fluorescent green (SYTO 9), whereas dead cells with damaged cell membranes and extracellular nucleic acids are stained with fluorescent red (SYTOX orange).

For CLSM samples, carbohydrates present in the EPS portion of *M. xanthus* ECM were stained with 5 µg/ml Alexa 633-conjugated derivatives of the wheat germ agglutinin lectin (Alexa 633-WGA, Invitrogen) in MOPS buffer [29]. Calcofluor white (Sigma, USA), a fluorescent dye binding EPS of *M. xanthus* [36], was used to stain the isolated cell-free extracellular materials at 5 µg/ml and visualized using fluorescent microscopy (Nikon E-400, Japan) with UV excitation.

FM 4-64 (Molecular Probes, USA), a lipophilic styryl compound that specifically binds to plasma membranes, was used at 5 µg/ml to observe the cell membranes of *M. xanthus* [37]. All specimens were incubated with the fluorescent dyes for 30 min at room temperature in the dark.

### Quantification of Staining Signals in CLSM Images

CLSM images of individual channels were exported as 8 bit uncompressed TIFF format, and the signals were quantified by the program COMSTAT as previously described (Heydorn *et al.*, 2000). An optimal threshold value was chosen and remained constant throughout the analysis of images for each sample.

### Quantitative Colocalization Analysis

CLSM images of SYTOX orange/Alexa 633-WGA stained samples were exported in 8 bit TIFF format and processed for colocalization analysis. The images were imported to JACoP (Fabrice P. Cordelieres, Institut Curie, Orsay, France), a plugin for the Image J software [38]. Pearson’s correlation coefficient (PCC), an overlap coefficient according to Manders (MOC), overlap coefficients M1 and M2, and an intensity correlation quotient (ICQ) were calculated as described previously [39], [40]. Five replicate experiments were conducted.

### DNA Binding Assay

DNA binding experiments were conducted with isolated EPS in combination with either chromosomal DNA from DK1622 or commercial salmon sperm DNA (2 Kb in size, 10 mg/ml, Invitrogen). 1 mg of isolated EPS was incubated with 0.1 mg chromosome DNA or salmon sperm DNA in 1 ml buffer at different pHs (0.15 M NaCl solution at pH 7.0; 0.1 M NaAc/HAc solution at pH 5.4; 0.01 M NaOH solution at pH 12.0), and denoted as ‘DNA+EPS’. *M. xanthus* strain SW504 is deficient in the EPS production due to an inframe deletion of *difA* gene [41]. 0.1 mg of DNA was incubated with 1×10^8^ SW504 cells in 1 ml of 0.15 M NaCl (pH 7.0) solution, and denoted as ‘DNA+Cell’. Samples containing SW504 cells were only tested at pH7. Solution containing DNA alone was used as control. The samples were mixed by vortexing for 15 sec, incubated at 32°C for 120 min, and centrifuged at 12,000×g for 30 min. 100 µl of the supernatant was transfered into fresh tubes. The DNA concentrations in the supernatants were determined with a Quant-iT high sensitivity DNA assay kit. The DNA binding percentage was calculated as the ratio of DNA bound to the pellet to the total DNA amount. Triplicate experiments were conducted.

### DNase I, SDS and Sonication Treatments of *M. xanthus* Starvation Biofilms

200 µg/mL DNase I (Worthington) was added to the system at the time of inoculation of biofilms and submerged cultures to remove eDNA. To assess the contribution of DNA to biofilm strength, 24 hr biofilms formed on the bottom of 12-well polystyrene culture plates were treated with DNaseI and then subjected to sonication (Sonicator S3000, Misonix, USA) at 60 W energy for 3 min or immersion for 1 hr in MOPS buffer containing 0.05% SDS. Crystal violet assays [22] were used to determine the relative biomass of the biofilms before and after sonication or SDS immersion. Biofilms without DNaseI treatment were used as controls for comparison. Triplicate experiments were conducted.

### Force-separation Curve Measurements

Atomic force microscopy (AFM) was employed to test the adhesive nanomechanical characteristics of DK1622 biofilms with or without DNase I treatment as described previously [42]. The force-separation curves were measured on a Bioscope II AFM system (Veeco Digital Instruments, USA) mounted on an inverted optical microscope (Carl Zeiss) using Si_3_N_2_ MSNL cantilevers (Veeco Digital Instruments) with experimentally measured spring constants of 0.03 N/m and a <10 nm tip radius. For different samples in the MOPS buffer at room temperature, the AFM tip was pressed against the biofilm surface with a force of ≈2 nN and subsequently retracted, and the force-separation curves were thus determined. All the force measurements using the contact mode were recorded at a pulling rate of 1 Hz.

## Results

### Isolated Cell-free Extracellular Materials from *M. xanthus* Contained both EPS and DNA

Our consistent observation that in *M. xanthus* DK1622 the DNA remained with the EPS portion during the extraction followed the standard protocol [15], triggered our curiosity to investigate a possible role of DNA in ECM formation. To rule out that the DNA was introduced by cell lysis during the EPS isolation process that includes EDTA and SDS treatments [15], [32], we developed a membrane-chamber system (Fig. 1A) that does not include cell damaging procedures to isolate cell-free extracellular materials from *M. xanthus* culture as described in *Materials and Methods*. Strain DK10547, a *gfp*-expressing derivative of DK1622 [35], was chosen to easily monitor the presence of cells in the lower chamber of the membrane-chamber system by visualizing GFP-fluorescence. After staining with calcofluor white and propidium iodide, both EPS and DNA were detected microscopically in the cell-free samples (Fig. 1B) collected in the lower chamber of the experimental setup with UV (Fig. 1C) and 543 nm excitation (Fig. 1D), respectively. Since this system allowed the separation of the cells from the extracellular materials (no cells were detected upon 488 nm excitation, data not shown) without artificially triggering cell lysis, the DNA detected in the collected samples could be a native component of ECM in *M. xanthus*.

### eDNA Formed Specific Structures in *M. xanthus* Biofilms

Next, we explored the nucleic acid distribution in *M. xanthus* submerged biofilms using confocal laser scanning microscopy (CLSM). With a mixture of SYTO 9 and SYTOX orange stains, extracellular as well as nucleic acids inside cells with compromised membranes were revealed by SYTOX orange labeling, while viable cells with intact membranes were stained by SYTO 9.

**Figure 2:**
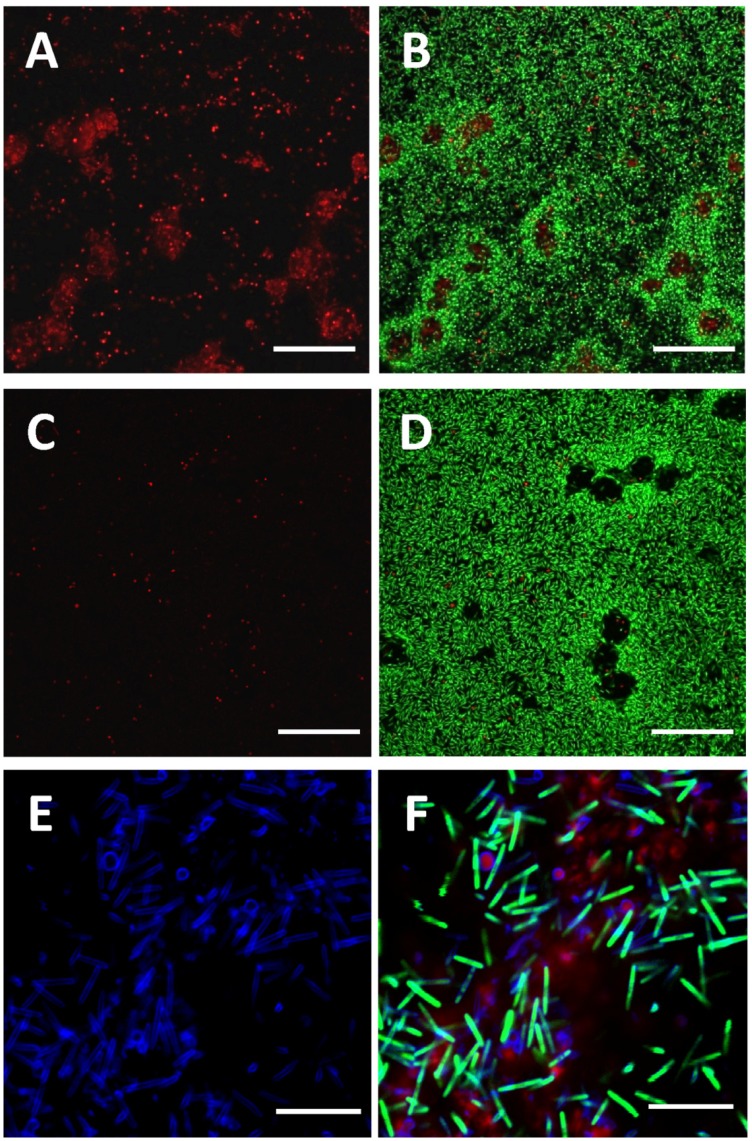
eDNA in *M. xanthus* non-developmental starvation biofilms. *M. xanthus* DK1622 biofilm structures formed in MOPS buffer at 24 hr (panels A and B) and DNase I treated biofilm (panels C and D) were counterstained with SYTOX orange (red) and SYTO9 (green). Panels A and C are the single channel images (SYTOX orange), and panels B and D are the overlaid images. *M. xanthus* DK10547 with a GFP label (green) formed starvation biofilms (panels E and F) in MOPs buffer at 24 hr was counterstained with SYTOX orange (red) and FM 4-64 (blue). Panel E is the single channel image (FM 4-64), and panel F is the overlaid image. The bars represent 50 µm in panels A–D and 10 µm in panels E and F.

In 24 hr non-developmental starvation biofilms, we observed both smeared and dot-like patterns for the SYTOX orange signals (Fig. 2A). In the overlaid image, the smeared structures were surrounded by live cells, while the dot-like patterns were dispersed throughout the biofilm (Fig. 2B). Furthermore, only the smeared signals disappeared after treatment with DNase I, and most of the dot-like signals remained intact (Figs. 2C and 2D). After quantification and statistical analyses of five samples, SYTOX orange signals in DNase I treated samples were about 0.6–4.3% compared to untreated samples. The smeared signals therefore likely result from SYTOX orange binding to eDNA molecules, which are exposed and thus sensitive to treatment with DNase I, rather than extracellular RNA. The dot-like signals might be nucleic acid molecules within cells with compromised membranes, whose DNA would be less accessible to treatment with DNase I. To precisely identify the location of eDNA in *M. xanthus* biofilms, a combination of SYTOX orange and the membrane-specific dye FM 4-64 was used to stain the starvation biofilm formed by *M. xanthus* DK10547 (a *gfp*-expressing derivative of DK1622). As shown in Figs. 2E and 2F, the red smeared structures stained by SYTOX orange (red) were confirmed as eDNA, which were not wrapped by the cell membrane (blue). The green signals (GFP) inside the blue membrane signals (FM 4-64) indicated live cells, and dead cells were identified by the red dot-like signals inside the blue spherical membrane signals.

**Figure 3:**
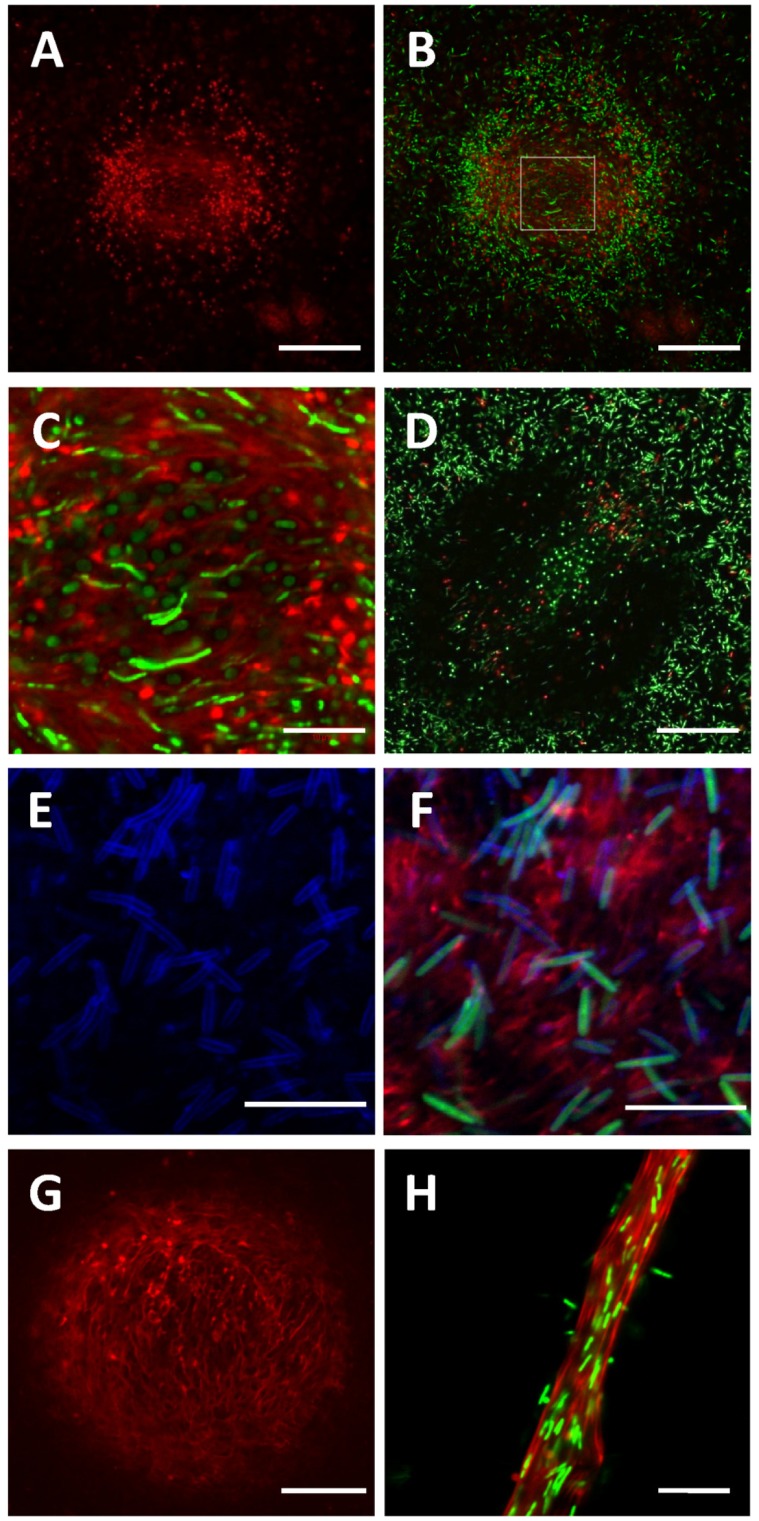
eDNA in *M. xanthus* submerged fruiting bodies. *M. xanthus* DK1622 fruiting body formed in submerged culture at 24 hr after starvation was initiated (panels A–C) and DNase I treated submerged fruiting body (panel D) were counterstained with SYTOX orange (red) and SYTO9 (green). Panels A is the single channel image (SYTOX orange), and panels B–D are the overlaid images. Panel C showed a magnified portion of panel B indicated by a white pane. *M. xanthus* DK10547 with a GFP label (green) formed fruiting body (panels E and F) in MOPs buffer at 24 hr was counterstained with SYTOX orange (red) and FM 4-64 (blue). Panel E is the single channel image (FM 4-64), and panel F is the overlaid image. Panel G shows a 48 hr submerged fruiting body of DK1622 stained with SYTOX orange, and panel H shows a trail structure in a 48 hr submerged culture of DK1622 stained with SYTOX orange and SYTO9. The bars represent 50 µm in panels A–D, 10 µm in panels E, F and H, and 20 µm in panel G.

More interestingly, well organized structures stained with SYTOX orange were observed in 24 hr submerged fruiting bodies (Fig. 3). The dot-like signals were distributed in a circular and symmetric manner surrounding the center of cell aggregation, while reticulated structures formed in the center of the initial fruiting bodies (Figs. 3A–3C). The red signals in DNase I treated samples were about 1.3–7.6% of untreated samples, and the reticulated structures were completely eliminated by the treatment (Fig. 3D), suggesting that they were exposed eDNA molecules. Some live cells were embedded in the reticulated structure of the eDNA (Figs. 3B and 3C). These observations were further confirmed by the examination of DK10547 fruiting bodies stained with SYTOX orange and FM 4-64 (Figs. 3E and 3F), which showed that the reticulated SYTOX orange signals (red) were not confined within plasma membranes (blue) and originated from the eDNA rather than from DNA molecules within dead or membrane-compromised cells. Furthermore, tighter eDNA structures were formed in the aggregation centers of 48 hr fruiting bodies and cell trails (Figs. 3G and 3H). To the best of our knowledge, this is the first report that eDNA forms specific structures rather than being randomly distributed in the ECM of *M. xanthus*.

### eDNA and EPS Colocalized in *M. xanthus* Biofilms

**Figure 4:**
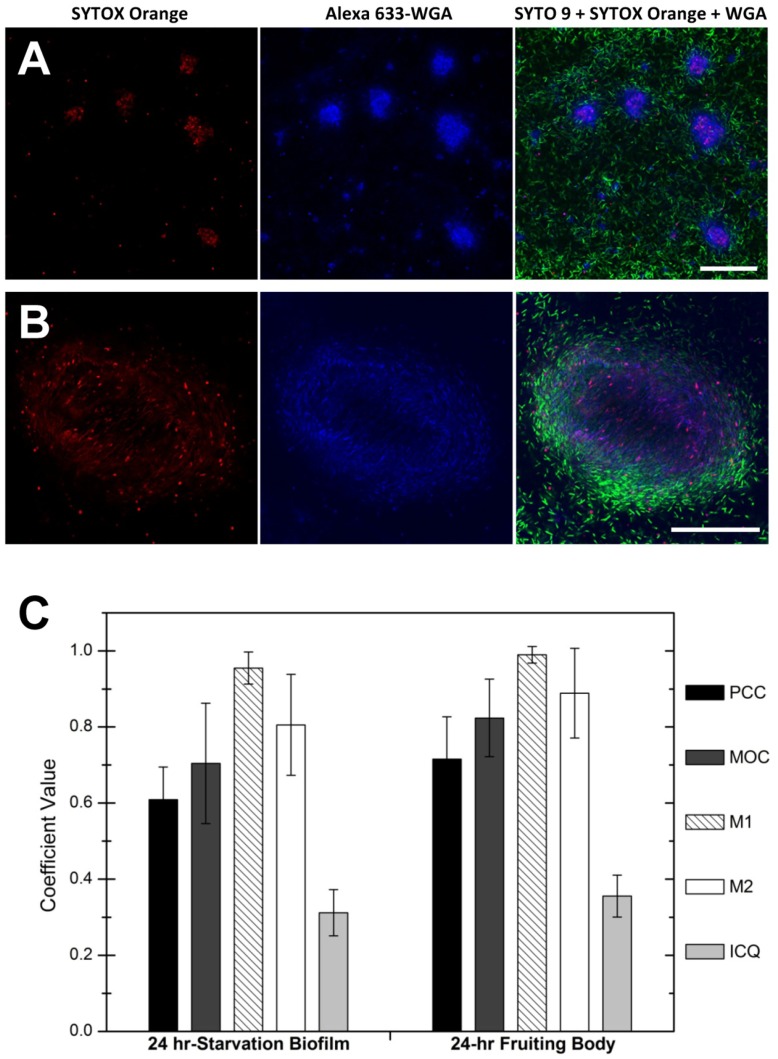
eDNA colocalized with EPS within *M. xanthus* starvation biofilm and fruiting body structures. Panel A, DK1622 starvation biofilm formed in MOPS buffer for 24 hr labeled with STYOX orange (red), Alexa 633-conjugated WGA (blue) and SYTO 9 (green). Panel B, DK1622 24 hr fruiting body structures with STYOX orange, Alexa 633-conjugated WGA and SYTO 9. Images in panel A were taken with a 40× objective using CLSM, and images in panel B were taken with a 63× objective. The bars in panels A and B represent 50 µM. Panel C showed the quantitative colocalization analysis results of STYOX orange (eDNA) and Alexa 633-WGA (EPS) signals from submerged 24 hr starvation biofilms (left) and 24 hr fruiting bodies (right). The PCC represents Pearson’s correlation coefficient, MOC represents overlap coefficients according to Manders, M1 represents colocalization coefficient M1 (fraction of eDNA overlapping EPS), M2 represents colocalization coefficient M2 (fraction of EPS overlapping eDNA), and ICQ represents intensity correlation quotient. Mean ± SD is plotted.

EPS is considered as the key component of the *M. xanthus* ECM and forms scaffolds within the fruiting bodies [15], [29]. Because a similar pattern of distribution was found for eDNA within fruiting bodies (Fig. 3), we utilized a combination of three fluorescent dyes to label biofilms (Fig. 4): STYO 9, STYOX orange and Alexa 633-conjugated WGA, a lectin that recognizes N-acetylglucosamine and was previously shown to specifically bind to the EPS of *M. xanthus* [29]. Under all conditions tested, eDNA always colocalized with EPS, which appears purple as a result of the overlaid red (mostly eDNA) and blue signals (EPS) (Figs. 4A and 4B). This observation was supported by the results of quantitative colocalization analysis. Upon examination of the respective PCC and MOC coefficients (0.6–0.9, Fig. 4C), the SYTOX orange and Alexa 633-WGA signals exhibited typical colocalization patterns [39], [43]. At the same time, the M1 coefficient indicated that a large proportion of the eDNA (SYTOX orange signal) coincided with the EPS (Alexa 633-WGA signal), and the M2 coefficients indicated a wider distribution of EPS in the intercellular space compared to eDNA (Fig. 4C). Next, an intensity correlation analysis of SYTOX orange/Alexa 633-WGA staining pairs was performed. The ICQ values of the staining pairs in both structures (Fig. 4C) were all in the range of 0–0.5 for the dependent staining [40] and thus significantly different from random staining (ICQ = 0; Student’s *t* test *p*<0.001; N = 5). This quantitative analysis was consistent with the CLSM observations. Furthermore, the WGA signals remained intact after treatment with DNase I (data not shown), ruling out the possibility that colocalization is a result of nonspecific binding of WGA to eDNA. The colocalization/co-existence of eDNA and EPS observed in both native ECM and isolated cell-free extracellular material provided evidence for potential direct interactions between eDNA and EPS in *M. xanthus*.

### DNA Bound to the EPS of *M. xanthus*

**Figure 5:**
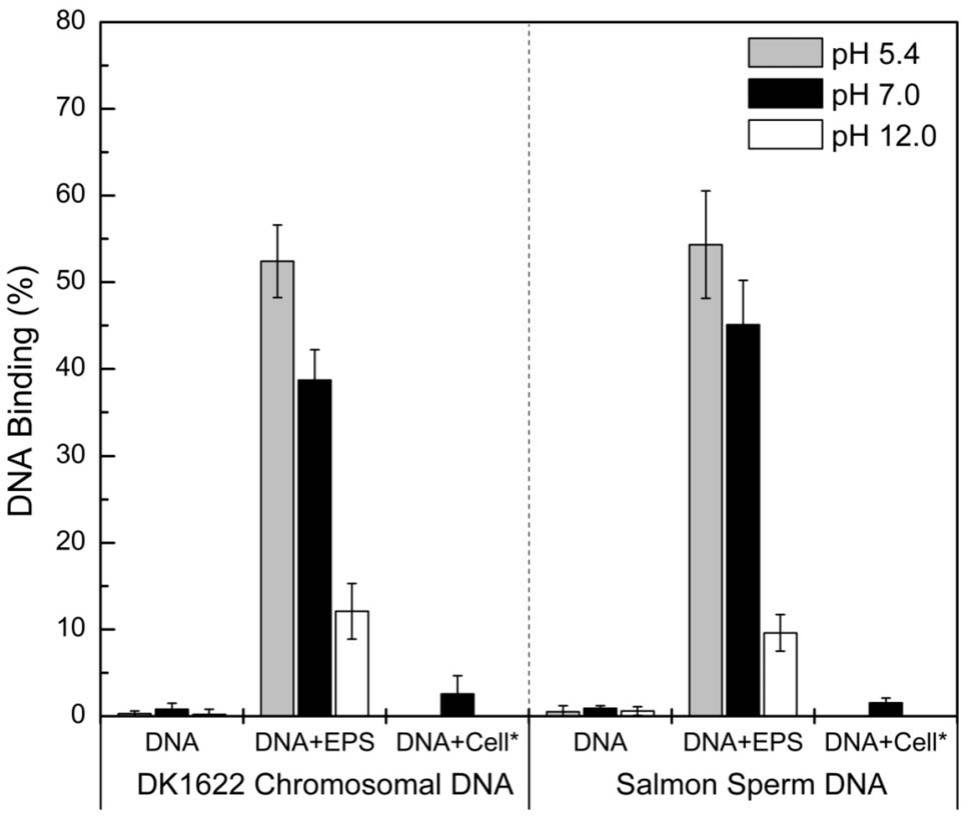
DNA bound to *M. xanthus* EPS at different pHs. The binding percentages of wild-type DK1622 chromosomal DNA (left) and commercial salmon sperm DNA (right) to isolated EPS were determined at different pHs, and the average ± SD is plotted. On the x-coordinate, ‘DNA’ represents different DNA samples, ‘EPS’ represents isolated *M. xanthus* EPS and ‘Cell*’ represents SW504 cells, which were added to the test system.

To further investigate the potential interactions between DNA and EPS, purified nucleic acid-free EPS was generated as described in *Material and Methods*. Significant binding to EPS was observed *in vitro* for both *M. xanthus* chromosomal DNA and salmon sperm DNA (Fig. 5) suggesting that the binding was independent of DNA sequence. We also explored the effect of pH on these interactions. At pH 7.0, about 40% of DK1622 chromosomal DNA or salmon sperm DNA bound to EPS, respectively (Fig. 5). Compared to the binding at pH 7.0, slightly higher DNA binding percentages were observed for both DNA samples at pH 5.4, while significantly lower binding percentages (Student’s *t* test *p*<0.01; N = 3) were observed at pH 12.0. These observations indicate a pH-dependent interaction between DNA and *M. xanthus* EPS. In order to test the DNA binding ability of *M. xanthus* cells, the EPS deficient mutant SW504 (*ΔdifA*) cells were mixed with DNA at pH 7.0, and less than 3% of DNA was precipitated by cells (Fig. 5). This result suggests that eDNA adheres to the cellular surfaces of *M. xanthus* via interactions with EPS.

### EPS-eDNA Interaction Increased the Mechanical Strength, Adhesion and Stress Resistance of the ECM

**Figure 6:**
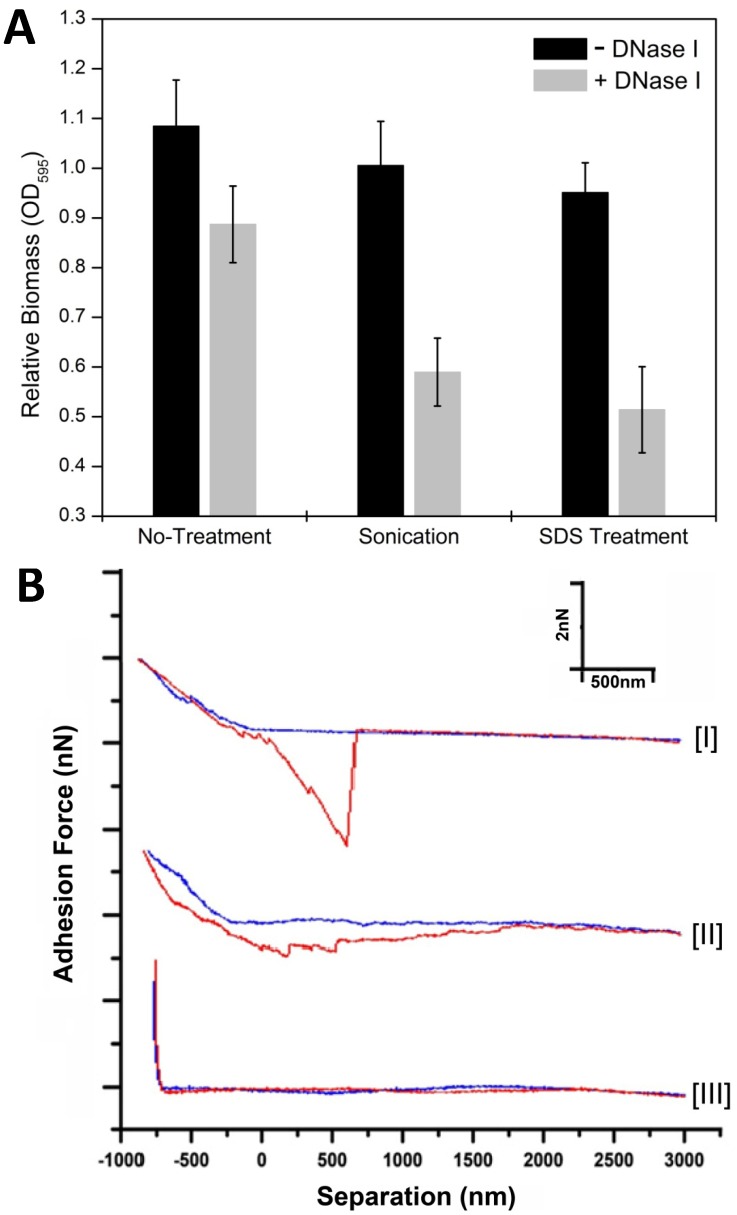
Mechanical strength, anti-disruptive properties and nanomechanical adhesive characteristics recorded using force-separation curves of the *M. xanthus* starvation biofilm matrix with or without eDNA. In panel A, DK1622 biofilms without DNase I (black bar) and with DNase I (grey bar) were established over a 24 hr time period in MOPS buffer and biomass was measured as crystal violet optical density (No-treatment control). The changes of biomass in these two kinds of biofilms after sonication or SDS treatment, respectively, are also shown. In panel B, representative force curves measured by AFM on DK1622 24 hr starvation biofilm matrix (curve I), matrix treated with DNase I (curve II) and bare portion of the substrate after the tip was used (curve III). Force-separation curves were recorded as “approach” (blue) and “retraction” (red) curves.

Having established that eDNA colocalized with EPS *in vivo* and bound to EPS *in vitro*, we further explored the biological significance of this interaction. We hypothesized that this binding could enhance the strength of the ECM and influence bacterial characteristics. Treatment of DK1622 24 hr starvation biofilms with DNase I resulted in higher sensitivity to both sonication and 0.05% SDS treatments (Fig. 6A), indicating a reduction in mechanical strength and resistance to detergents. At the same time, the DNase I treatment itself reduced the biomass of biofilms to some extent, suggesting the involvement of eDNA in the architectural features of *M. xanthus* starvation biofilm.

The force-spectroscopic capacity of atomic force microscopy (AFM) was employed to investigate the nano-mechanical properties of native *M. xanthus* matrices [42] subjected to DNase I treatment in comparison to untreated samples. The force-separation curves were measured on different samples under physiological buffer conditions by pressing the AFM tip against the biofilm surface with a force of ≈2 nN and subsequently retracting the tip. Several rupture events were recorded, arising from the breakage of multiple adhesions between the tip and ECM (Fig. 6B-Curves I and II). The force curve was also measured on bare substrate after measurement on ECM to confirm that the tip was not contaminated with matrix material that would introduce artifacts (Fig. 6B-Curve III). DK1622 with native ECM showed a higher degree of surface adhesion than eDNA-free ECM. We measured about 50 different spots on each type of matrix and the average adhesive force obtained on untreated samples was 1.8±0.7 nN, much greater than forces obtained on samples treated with DNase I (0.35±0.21 nN). At the same time, the adhesive events on the untreated samples usually ceased after ≈1 µm of tip retraction (Fig. 6B-Curve I), while the treated samples displayed an extended retraction length of ≥2 µm, including several minor adhesive events (≈0.1 nN). These findings showed that the biofilms treated with DNase I were less compact in structure and extended more into the solution compared to the untreated samples.

## Discussion

Although the presence of eDNA in the ECM of *M. xanthus* has been proposed previously [16], little is known about its distribution and roles. In this study, we provide evidence that eDNA forms specific structures in *M. xanthus* biofilms, such as smear patches in the non-developmental starvation biofilms (Fig. 2) and reticulated structures in fruiting bodies (Fig. 3). Furthermore, the finding that eDNA and EPS co-localized in the ECM (Fig. 4) prompted us to explore the mechanism of eDNA involvement in *M. xanthus* biofilm formation and its interaction with EPS molecules. In certain other bacterial biofilms, eDNA also forms organized structures [24], [44], [45], [46] and is related with EPS in some cases [26], [47]. In *Pseudomonas aeruginosa*, eDNA is required for the formation of biofilms [22] and concentrated among dead cells in the Psl exopolysaccharide matrix-free area of biofilms [47]. Biofilms of *Streptococcus pneumonia* also contain eDNA that is partially associated with EPS; though the eDNA is often excluded from the thickest parts of biofilms and does not form specific structures [26]. Little is known regarding the potential interactions between eDNA and other macromolecules in the establishment of biofilms.

Our results indicate that in *M. xanthus*, the colocalization and co-distribution of eDNA and EPS are attributed to their direct binding (Fig. 5). Only slight differences between two DNA molecules, DK1622 chromosomal DNA and commercial salmon sperm DNA, in EPS binding were observed, suggesting the DNA-EPS interaction was not dependent on the size and origin of the DNA. The DNA-EPS interaction is clearly influenced by pH, which is similar to the reported pH-induced DNA association with chitosan (composed of N-acetyl-glucosamine and glucosamine monomer units) [48]. Glucosamine has been identified in the *M. xanthus* EPS [15], and could act as the cationic moieties of EPS that interact with anionic DNA molecules. For many well characterized EPS structures, computer modeling has revealed that charged groups are typically on the exterior of molecular chains and can readily interact with other charged molecules [10] such as DNA. In general, three types of weak forces mediate the interactions between macromolecules in ECM: dispersion forces, electrostatic interactions and hydrogen bonds [49]. We suggested that electrostatic forces could play important roles in the polymer-polymer interactions between DNA and *M. xanthus* EPS. As far as we know, this is the first demonstration that the distribution of eDNA is correlated with EPS through direct interactions in bacterial biofilms and this also provides novel insight into eDNA integration into biofilm.

Since EPS is the key component of *M. xanthus* ECM [15], [29], we hypothesized that the role of eDNA in *M. xanthus* was to enhance matrix stability. Through *in situ* force spectroscopy, we were able to perform quantitative nanomechanical characterization of the *M. xanthus* biofilm matrix revealing the mechanical nature of ECM adhesion (Fig. 6B). Native ECM containing both DNA and EPS exhibited stronger adhesive strength and more compactness compared to the ECM without DNA. This could contribute to differences in the initial adhesion processes of the biofilm where the native matrix could lead to a more efficient biofilm settlement than the one treated with DNase I. Furthermore, the integrated extracellular matrix conferred additional stress resistance against sonication and SDS treatments (Fig. 6A), suggesting that the presence of eDNA and its interaction with EPS is highly relevant to strength of biofilm ECM and survival under adverse environments. In addition, EPS has been shown to be an extracellular barrier and able to block the plasmid transformation in *M. xanthus* [50]. This may occur through the EPS interaction with eDNA which would lower DNA local effective concentration, thus allowing only cells deficient in EPS production to be naturally transformable. These findings support the idea that the interactions between eDNA and EPS play a number of roles in the sophisticated cell behaviors of *M. xanthus*.

There is still much debate over the source of bacterial biofilm integrated eDNA, though several possibilities have been proposed [1]. For example, eDNA could be derived from lysed cells [51], [52], [53], released via small vesicles from the outer membrane of cells [54], [55] or actively secreted [1], [56], [57], [58]. In *M. xanthus*, it has been presumed that the eDNA present in the ECM is generated by developmental autolysis of cells [16]. The reported amounts of autolysis in *M. xanthus* has varied from as little as 20% to as 80–90% [59], [60]. Our experiments confirmed the presence of dead cells in *M. xanthus* biofilms, which could contribute to the eDNA in ECM. Considerable amount of eDNA were observed both in the simple non-developmental starvation biofilms and the highly-organized developmental fruiting bodies, in spite of the developmental autolysis was mostly occurred in the fruiting bodies. Therefore, it remains unclear if the cell autolysis is the only source of eDNA in *M. xanthus* biofilms. If eDNA in *M. xanthus* was only derived from cell lysis, it would be expected that eDNA is spatially associated with dead cells, as in *P. aeruginosa* biofilms [47]. Moreover, in the mutants lacking programmed cell death, such like *ΔmazF* [61], the amount of eDNA should be dramatically decreased.

In summary, our results demonstrate that during the establishment of *M. xanthus* biofilms, eDNA is recruited by the EPS and integrated into the ECM by binding to EPS components. This allows establishment of a biofilm scaffold with a strong matrix which conveys increased resistance to environmental stresses. This study provides further understanding of the DNA-EPS matrix and presents *M. xanthus* biofilms as an intriguing model for exploring DNA-EPS interactions in bacteria. These findings also illustrate an unconventional role for DNA, expanding its list of potential cellular functions achievable through its molecular structure.
